# ROS and radiotherapy: more we care

**DOI:** 10.18632/oncotarget.16613

**Published:** 2017-03-27

**Authors:** Pierre Sonveaux

**Affiliations:** Pole of Pharmacology, Institut de Recherche Expérimentale et Clinique, Université Catholique de Louvain, Brussels, Belgium

**Keywords:** radiotherapy, tumor metabolism, reactive oxygen species, thioredoxin, auranofin

Cancer cells in tumors *in situ* constantly thrive to survive and proliferate in a microenvironment influenced by the nature of the tissue of origin, the presence and activities of host cells, vascular and lymphatic density and dynamics, and often characterized by inadequate nutrient and oxygen supply and suboptimal waste removal. Successful cancer cells are those that succeed to exploit at best their metabolic possibilities and are effective to rapidly rewire metabolic pathways in function of external influences and internal needs. External influences comprise anticancer treatments that cancer cells can combat with adapted metabolic solutions. A first example is related to chemotherapy: because most chemotherapeutic drugs are weak bases, the production and secretion of lactic and carbonic acids as well as lysosomal acidification provide pH barriers preventing optimal transmembrane drug delivery and accounting for lysosomal drug storage/inactivation [1]. A second example was recently provided with anti-angiogenic therapies: cancer cells can adapt to a low vascular density by engaging a metabolic symbiosis between glycolytic cells that consume glucose and release lactate and oxidative cells that consume lactate preferentially to glucose [2]. A third example relates to radiotherapy. It is indeed well known that local availability of molecular oxygen enhances the efficacy of radiotherapy, as DNA lesions caused by reactive oxygen species (ROS) produced during water radiolysis react with oxygen to from stable DNA peroxides [3]. Thus, cancer cells that receive less or consume more oxygen are typically more radioresistant than well oxygenated cancer cells consuming little oxygen. Consequently, tumor perfusion and cancer cell respiration can be manipulated for radiosensitization [4].

The study by Wang H *et al*. [5] in this issue of *Oncotarget* constitutes a particularly good example of how cancer cells can exploit their metabolic activities to resist to radiotherapy, and of what can be done therapeutically to counteract such intrinsic radioresistance. As metabolic entities, cancer cells are deemed to reach three objectives: the production of enough energy for cell proliferation and repair (bioenergetics), the generation of building blocks for the same purposes (biosynthesis), and the maintenance of a pool of reductive molecules that protect against redox stress [6]. The latter component is particularly relevant to radiotherapy and to some forms of chemotherapy (*e.g*. anthracyclines) that precisely kill cancer cells by causing a redox imbalance in favor of excessive oxidation [4, 7]. Thus, these therapies display full anticancer power only when they overcome the redox defenses of cancer cells, and cancer cells can resist to redox insults by boosting antioxidant defenses.

Glutathione (GSH) and thioredoxin (Trx) are the two major endogenous antioxidant peptides. Their production tightly depends on protein biosynthesis (in particular glutamine metabolism for GSH production) and their redox recycling on the production of NADPH that donates electrons to glutathione reductase (GR) and thioredoxin reductase (ThxR). In cancer cells, NADPH mostly originates from the oxidative arm of the pentose phosphate pathway (a glucose-dependent pathway) and from the malic enzyme converting malate exported from mitochondrial tricarboxylic acid (TCA) cycle to pyruvate in the cytosol (a pathway that can be fueled by several substrates, including glutamine and lipids) [6].

In the context of X-ray radiotherapy, Wang H *et al*. [5] demonstrate experimentally that Auranofin, an antirheumatic agent, could be repurposed as a radiosensitizer in cancer. Auronafin is indeed a potent ThxR inhibitor that acts by forming a three-coordinate intermediate gold(I)-selenolate complex, thus blocking the activity of the selenoenzyme [8]. Using mouse mammary carcinoma cells as model, Wang H *et al*. [5] report that low micromolar doses of Auronafin that are not cytotoxic *per se* disarm cancer cells against redox insults, which the authors exploited with radiotherapy. The combination strategy was effective in both hypoxic and normoxic cancer cells, demonstrating that a high ThxR activity is an essential component supporting intrinsic radioresistance. The authors further identified that Auronafin alters mitochondrial activities in cancer cells, resulting in oxygen sparing for radiotherapy. Best observed radiosensitization was achieved when combining Auronafin with buthionine sulphoximine, a GR inhibitor.

While precise dose deposition to the tumor can be achieved with highly pursued technological improvements of radiotherapy thus limiting side effects to healthy tissues, radioresistance comparatively becomes a prevalent concern. The importance of the findings of Wang H *et al*. [5] is that the radiosensitizing strategy that they identified is expected to be rapidly implemented in clinical practice with the repurposing of FDA-approved drug Auranofin from an antirheumatic to a radiosensitizing agent.
